# Is ultrasound axillary staging less accurate in invasive lobular breast cancer than in ductal breast cancer?

**DOI:** 10.1186/bcr3251

**Published:** 2012-11-09

**Authors:** P Sankaye, G Porter, J Steel, S Doyle

**Affiliations:** 1 Plymouth Hospitals NHS Trust, Plymouth, UK

## Objective

To identify whether axillary US is less accurate in invasive lobular breast cancer than in ductal breast cancer.

## Methods

Randomised cohorts of screening and symptomatic patients were retrospectively identified from histology records of 2010/11. Axillary US of 65 patients with primary breast cancers (BC) from each group of invasive lobular cancer (ILC) and invasive ductal cancer (IDC) were reviewed. Preoperative US-guided needle biopsy sampling was performed on abnormal lymph nodes (LN).

## Results

See Tables 1 and 2.

**Table 1:** 

	IDC	ILC	*P *value
Node-positive BC	34% (22/65)	32% (21/65)	
Preoperative US detection of node-positive disease	54% (12/22)	52% (11/21)	0.5
Abnormal US LN appearance	37% (24/65)^a^	26% (17/65)^b^	0.2
False negative rate for axillary US biopsy	18% (4/22)	12% (2/16)	0.5

^a^One unable to biopsy due to technical factors, one failure to sample node-B1. ^b^One failure to sample node-B1.

**Table 2:** 

	IDC	ILC
US sensitivity	0.73(0.51 to 0.88)	0.73(0.48 to 0.89)
US specificity	0.83 (0.68 to 0.92)	0.93 (0.8 to 0.98)
US positive predictive value	0.70 (0.48 to 0.86)	0.82 (0.55 to 0.95)

There were no statistically significant differences between the two groups.

## Conclusion

The previous literature on this topic is inconclusive. Some authors have suggested axillary ultrasound in ILC may be less accurate than in IDC, with a higher false-negative axillary assessment rate. Another study concluded that axillary US accuracy rates in ILC were comparable with previous published studies of IDC, used FNA in all cases. We specifically compared accuracy rates of preoperative axillary staging between ILC and IDC in own institution, with 14G needle biopsy as the procedure of choice to sample abnormal nodes. We found that there is no statistical difference in accuracy in US axillary staging between ILC and IDC.

